# Supplementary material for: Topographic changes in macula and its association with visual outcomes in idiopathic epiretinal membrane surgery
Source: PLoS One. 2025 Jan 9;20(1):e0316847. doi: 10.1371/journal.pone.0316847 (PMC11717306; doi:10.1371/journal.pone.0316847)
Supplement: Attachment — Submitted filename: Peer Review.pdf [file pone.0316847.s002.pdf]

Peer Review Report

Summary of the Manuscript:

The manuscript explores the macular topographic changes and their association with visual acuity changes and metamorphopsia in patients with idiopathic epiretinal membranes (iERM). This was executed through a retrospective chart review of 24 eligible consecutive patients who underwent iERM peel and were evaluated over the course of 6 months post-operatively. The authors aim to determine whether multimodal imaging of fundus photographs, optic coherence tomography (OCT) and OCT-angiography (OCT-A) may be of prognostic value regarding visual acuity and improvement of metamorphopsia.

Overall Evaluation:

This study provides valuable information on how changes in anatomical findings of the macula can help us in having an idea about what to expect in terms of visual prognosis after ERM peel surgery. The overall methodology is a significant strength of the study, but there are several areas where the manuscript could be improved, as mentioned below:

Comments:

1. **Introduction:**
    - The manuscript's introduction successfully provided background information on iERM as it elaborated on associated exam findings, visual outcomes and symptoms. However, it needs to better explain that the aim was to compare the macular topography changes, visual acuity and metamorphopsia pre-operatively versus post-operatively as part of establishing prognosis (as depicted in the title and conclusion).
2. **Results:**
    - Statistics in tables 1 through 4 have been reported as mean +- SD; range. However, non-parametric tests were used for further data analysis, meaning that the data obtained was not normally distributed. A better way to present central tendency and measures of dispersion in that case is median (Q1, Q3) or median [min-max].
    - In table 1, baseline BCVA is reported as a mean of 0.65 +- 0.25. In the text below it, it is said that baseline BCVA significantly improved from 0.42 +- 0.22 at baseline to 0.12 +- 0.15 at 6 months. Which value is the correct baseline BCVA?
3. **Discussion:**
    - The discussion does highlight the important points to be covered, but more concrete findings and examples should be given when the authors mention that many studies looked at topographic changes in the macula after surgery but did

not correlate them with BCVA due to conflicting findings regarding the methodology.

4. **Clarity and Structure:**
   - Regarding the methodology behind the calculation of the fovea-disc and fovea-arcades distances, I would suggest to briefly explain the steps done in order to do so and then mention that further details are shown in Figure 1 instead of the other way around, as the legend gives a great stepwise explanation on how this was done.
   - Figures 1 and 3 are a little blurry. Higher quality figures would make them easier to navigate, particularly for figure 1 as the edges of the optic disc are important landmarks to the methodology behind the study.

Minor Comments:

- Introduction, page 1: "the gradual distortion of the retinal structure" can be replaced with "the gradual distortion of the retinal layers."
- Introduction, page 1: the words "most" and "have" in the sentence "However, most studies have not used multimodal imaging" could be replaced with the words "previous" and "did", respectively.
- Introduction, page 1: the sentence "This methodological weakness may contribute to the current discrepancy in the prognostic values of some factors" is ambiguous. It would be a good idea to rephrase it to explain the fact that previously studied methodologies failed to account for confounding variables with ERM-affected eyes that might affect prognosis of surgery. The example that follows that sentence illustrates the idea really well.
- Materials and methods, study participants, page 3: add the words "with an" to the sentence "Eyes presenting (<u>with an)</u> idiopathic epiretinal membrane that typically covered the fovea"
- Materials and methods, study participants, page 3: add a dash between the words "age" and "related" to describe age-related macular degeneration.
- Material and methods, Ocular examination and Imaging, page 4: add the words "measurement" and "assessment of" for better clarity: "All patients underwent comprehensive ophthalmologic examination at baseline and the 1-, 3- and 6-month follow-up visits, including best-corrected visual acuity (BCVA) (<u>measurement</u>), (<u>assessment of</u>) metamorphopsia."
- Material and methods, Ocular examination and Imaging, page 5: add the word "also" in the sentence "Fundus photography, OCT, and OCTA were (<u>also</u>) performed on the healthy fellow eyes".
- Material and methods, main outcomes measures and statistical analysis, page 6: replace "for normality tests" with "to test for normality" in "Kolmogorov-Smirnov and Shapiro-Wilk analyses were used for normality tests."
- Material and methods, main outcomes measures and statistical analysis, page 7: replace "the factors" with "variables" in "Binary logistic regression was used to analyze the factors associated with visual outcomes."

- Results, page 8: add the word “present” in the sentence “The EIFL was (present) in 7 eyes.”
- Table 1: it is visually better to present sex and lens status as proportions using percentages instead of solely reporting absolute numbers.
- Table 1: It should be mentioned what the measures used for the symptom period are i.e. (mean +-SD, range).
- Table 1: A typo in the word "months" (misspelled as "month") next to “symptom period” should be corrected.
- Results section, under “Topographic changes in the Macula”, first sentence, page 9: the terms “the changes in parameters” and “their associations” are ambiguous. It would be helpful to the reader to define what those entail.
- Results, page 9, in Fig. 2 legend: “the X coordinate moved to the disc after 6 months” could be replaced with “the X coordinate moved towards the disc after 6 months.”
- Figure 2 legend, page 9: I suggest that the last 2 sentences of C and D be included as part of the results section of the manuscript and not as part of the legend, as they describe findings. I would also just describe what the graphs are about in (A) and (B) and explain the findings in the manuscript.
- Results, page 10: could replace the word “to” with “towards” in the sentence: “the fovea continuously moved to the optic disc during the follow-up period.”
- Results, page 11, figure 3 legend: remove the dash in “blue-cross” and “red-cross”.
- Results, page 11, figure 3 legend: remove the dash in “blue-cross” and “red-cross”.
- Results, page 11, figure 3 legend: could remove the last sentence as this was already mentioned in the results and will be reiterated in the discussion.
- Results, bottom of page 11, under Association between topographic factors and visual outcomes: add the word “with” in the sentence: “eyes with a thicker CSMT presented (with) worse BCVA at baseline.”
- Results, bottom of pages 11 and 12, under Association between topographic factors and visual outcomes: “change in BCVA” and “change in metamorphopsia” should be subtitles for better clarity.
- Results, page 13: replace “such” with” “those” in “Such eyes still showed worse vM and hM scores”
- Discussion, second sentence, page 14: “the fovea moved horizontally to the optic disc” could be replaced with “the fovea moved horizontally towards the optic disc”, implying that it moved nasally, as the results described.
- Discussion, page 14, last sentence of first paragraph can be rewritten as follows: “This study investigated the association between macular topographic changes and visual acuity via multimodal imaging comprising fundus photographs, OCT, and OCTA.”
- Discussion, page 15: A typo in the word "INL" (misspelled as "ILN") in the sentence “ILN thickness on OCT” should be corrected.
- Discussion, page 16: A typo in the word "disc" (misspelled as "disk") in the sentence “more horizontal movement of the fovea to the optic disk” should be corrected.

- Discussion, page 16: “more horizontal movement to the optic disc” could be replaced with “more horizontal movement towards the optic disc.”
- Discussion, page 16: “foveal movement to the optic disc” could be replaced with “foveal movement towards the optic disc.”
- Discussion, page 16: add the terms “in the context of” for better clarity in the sentence: “This reasoning about metamorphopsia have also been suggested (in the context of) macular hole.”
- Discussion, page 17: can rephrase “Different outcomes can be achieved if eyes with iERM are classified and distinguished based on severity” to “We may also obtain different outcomes if eyes with iERM were classified and distinguished based on severity” for better flow of the paragraph.
- In figure 1 (E), the text is not very clear.
- In figure 1, I would make the red and blue crosses more apparent (i.e. more bold and sharper borders). I would also consider changing the color of the red cross to something with higher contrast as red blends in with the color fundus photo’s background.
- In figure 3, I would suggest replacing the red dot at the optic disc with another color such a black or green as red might be mistaken as part of retinal blood vessels.
- In figure 3, make the blue cross more apparent in (A) and the red cross more apparent in (B). Again, in (B) and (C), I would also suggest changing the color of the red cross for the same reason discussed above for figure 1.
