# Supplementary material for: Topographic changes in macula and its association with visual outcomes in idiopathic epiretinal membrane surgery
Source: PLoS One. 2025 Jan 9;20(1):e0316847. doi: 10.1371/journal.pone.0316847 (PMC11717306; doi:10.1371/journal.pone.0316847)
Supplement: Attachment — Submitted filename: Response to Reviewers.docx [file pone.0316847.s003.docx]

**Point-by-point response to the reviewer’s comments**

Dear Editor & Reviewers,

We appreciate all suggestions and corrections. All authors have carefully reviewed these comments. Our detailed responses to comments are addressed below.

**Review Comments to the Author**

**Reviewer #1**: Notes:

**1.Change**: “The idiopathic epiretinal membrane (iERM) commonly appears in older people as glistening amorphous membranes on the macula” to “Idiopathic Epiretinal membrane commonly appears in the older population as a glistening amorphous membrane on the macula”

**Response 1:** As the reviewer’s comments, the corresponding sentence has been corrected **(First sentence of introduction)**.

**2. Would also include in the references about previous studies the following study:** Vingopoulos F, Koulouri I, Miller JB, Vavvas DG. Anatomical and Functional Recovery Kinetics After Epiretinal Membrane Removal. Clin Ophthalmol. 2021;15:175-181
<https://doi.org/10.2147/OPTH.S264948>

**Response 2:** The recommended reference was added according to the reviewer’s comment **(Line 9 of the second paragraph in the introduction)**.

**Reviewer #2**: Peer Review Report (also attached)
Summary of the Manuscript:
The manuscript explores the macular topographic changes and their association with visual acuity changes and metamorphopsia in patients with idiopathic epiretinal membranes (iERM). This was executed through a retrospective chart review of 24 eligible consecutive patients who underwent iERM peel and were evaluated over the course of 6 months post-operatively. The authors aim to determine whether multimodal imaging of fundus photographs, optic coherence tomography (OCT) and OCT-angiography (OCT-A) may be of prognostic value regarding visual acuity and improvement of metamorphopsia.

Overall Evaluation:
This study provides valuable information on how changes in anatomical findings of the macula can help us in having an idea about what to expect in terms of visual prognosis after ERM peel surgery. The overall methodology is a significant strength of the study, but there are several areas where the manuscript could be improved, as mentioned below:
Comments:
1. Introduction:
o The manuscript’s introduction successfully provided background information on ERM as it elaborated on associated exam findings, visual outcomes and symptoms. However, it needs to better explain that the aim was to compare the macular topography changes, visual acuity and metamorphopsia pre-operatively versus post-operatively as part of establishing prognosis (as depicted in the title and conclusion).

**Response:** As comments of reviewer 1 and 2, we revised the introduction.

2. Results:
o Statistics in tables 1 through 4 have been reported as mean +- SD; range. However, non-parametric tests were used for further data analysis, meaning that the data obtained was not normally distributed. A better way to present central tendency and measures of dispersion in that case is median (Q1, Q3) or median [min-max].

**Response:** In agreement with the reviewer’s opinion, the data in the results and tables 1 through 4 were changed to median and interquartile range.

In statistical analysis, “Continuous variables not normally distributed were expressed as median and interquartile range.” was added.

o In table 1, baseline BCVA is reported as a mean of 0.65 +- 0.25. In the text below it, it is said that baseline BCVA significantly improved from 0.42 +- 0.22 at baseline to 0.12 +- 0.15 at 6 months. Which value is the correct baseline BCVA?

**Response:** The correct figure was 0.42 +- 0.22 which had been presented in the text. There appears to have been a typo. Expressed as median and interquartile range, it was changed to 0.40 (interquartile range, 0.23 – 0.50) **(Results, Table 1).**

3. Discussion:
o The discussion does highlight the important points to be covered, but more concrete findings and examples should be given when the authors mention that many studies looked at topographic changes in the macula after surgery but did not correlate them with BCVA due to conflicting findings regarding the methodology.

**Response:** In the third paragraph of the Discussion, various studies that investigated tangential contraction and the characteristic research methods for each study are described as follows.

“The tangential changes of the macula were assessed using various methodologies as follows: the nasal area and angle between the inferior and superior vascular arcades, the vessel segment length and movement in a particular region, the distance between vascular arcades across the fovea, the distance from the fovea to the small retinal vessels around the fovea, and the distances between the intersections of retinal vessels around the fovea“.

In addition, it was stated that most of these studies without multimodal method failed to determine the relationship between visual acuity and topological change (“However, many of them did not confirm the association between the changes in topographic parameters and BCVA following surgery”).
In the sentence “Most studies observed that significant changes in their topographic parameters were associated with the OCT parameter of macular thickness.”, the expression “Most studies” had been changed to “Most of these studies” to emphasize the connection with the previous sentence.

4. Clarity and Structure:
o Regarding the methodology behind the calculation of the fovea-disc and fovea arcades distances, I would suggest to briefly explain the steps done in order to do so and then mention that further details are shown in Figure 1 instead of the other way around, as the legend gives a great stepwise explanation on how this was done.

**Response:** In the methods, the description of techniques used to measure variables had been changed briefly as follows:

from “First, we placed three dots at the junctions of the large retinal vessels and optic disc margin to serve as landmarks. We then superimposed the baseline and corresponding follow-up fundus photographs to confirm their concurrence and minimize the mismatch of features between the two photographs [23]. Second, the foveal center was identified on the superficial capillary plexus map of the *en face* OCTA images. If there was no foveal avascular zone (FAZ), the marker was located at the point where the outer nuclear layer was the thickest and the inner nuclear layer was absent or thinnest, using sectional OCT images of the 3D macular cube mode. The OCTA images were transformed and overlapped by matching the vasculature using a customized program, and the foveal center was marked on the corresponding fundus images.” to “First, baseline and follow-up photographs were superimposed using three dots at the junctions of the large retinal vessels and optic disc margin [23]. Second, the foveal center was identified on the superficial capillary plexus map of the *en face* OCTA images. If there was no foveal avascular zone (FAZ), the marker was located at the point where the outer nuclear layer was the thickest and the INL was absent or thinnest, using sectional OCT images of the 3D macular cube mode. Using a customized program, the foveal center identified in OCTA images was marked on the corresponding fundus images.” **(Materials and methods, Distance of Fovea-Disc and Fovea-Vascular Arcades and Foveal Location, 1^st^ paragraph, 2^nd^ sentence).**

o Figures 1 and 3 are a little blurry. Higher quality figures would make them easier to navigate, particularly for figure 1 as the edges of the optic disc are important landmarks to the methodology behind the study.

**Response:** As the reviewer mentioned, the positioning of the landmarks on the edges of the optic disc was most important in this study and could have the greatest impact on reproducibility. Fig 1 presents the methodology using multimodal images as it was actually carried out. The reason Fig 1a is blurred is because each image has been changed to transparent with low opacity to overlap the two images. Small, illegible letters in Fig 1e have been removed. The blurry parts in Figure 3C have been somewhat ameliorated. However, since the image has been greatly enlarged, there are limitations in improving the blurry parts due to problems with the original image quality. Due to the large size of the figure file, there was a slight deterioration in image quality after image processing using PACEV provided by PLOS ONE, but considering the nature of the electronic journal, accurate structures and markers can be confirmed by viewing the uploaded original image rather than printed size image. **(Figure 1 and 3)**

Minor Comments:

o Introduction, page 1: “the gradual distortion of the retinal structure” can be
replaced with “the gradual distortion of the retinal layers.”

**Response:** The corresponding sentence has been corrected according to the reviewer’s comment **(Introduction, 1^st^ paragraph, 6^th^ line)**
o Introduction, page 1: the words “most” and “have” in the sentence “However, most studies have not used multimodal imaging” could be replaced with the words “previous” and “did”, respectively.

**Response:** The corresponding sentence has been corrected according to the reviewer’s comment **(Introduction, 3^rd^ paragraph, 1^st^ line)**
o Introduction, page 1: the sentence “This methodological weakness may contribute to the current discrepancy in the prognostic values of some factors” is ambiguous. It would be a good idea to rephrase it to explain the fact that previously studied methodologies failed to account for confounding variables with ERM-affected eyes that might affect prognosis of surgery. The example that follows that sentence illustrates the idea really well.

**Response:** The corresponding sentence has been corrected according to the reviewer’s comment: From “This methodological weakness may contribute to the current discrepancy in the prognostic values of some factors (e.g., various factors influencing each other in eyes with iERM, which can be confounding factors in predicting prognosis).” to “Various factors influence each other in eyes with iERM, which can be confounding factors in predicting prognosis. Previously studied methodologies were hard to figure out confounding variables that might affect prognosis of surgery.”. **(Introduction, 3^rd^ paragraph, 2^nd^ sentence).**

o Materials and methods, study participants, page 3: add the words “with an” to the sentence “Eyes presenting (with an) idiopathic epiretinal membrane that typically covered the fovea”

**Response:** The corresponding sentence has been corrected according to the reviewer’s comment **(Materials and methods, study participants, 2^nd^ paragraph, 2^nd^ sentence).**
o Materials and methods, study participants, page 3: add a dash between the words “age” and “related” to describe age-related macular degeneration.

**Response:** The corresponding sentence has been corrected according to the reviewer’s comment **(Materials and methods, study participants, 2^nd^ paragraph, 13^th^ line).**
o Material and methods, Ocular examination and Imaging, page 4: add the words “measurement” and “assessment of” for better clarity: “All patients underwent comprehensive ophthalmologic examination at baseline and the 1-, 3- and 6- month follow-up visits, including best-corrected visual acuity (BCVA) (measurement), (assessment of) metamorphopsia.”

**Response:** The corresponding sentence has been corrected according to the reviewer’s comment **(Materials and methods, Ocular Examination and Imaging, 1^st^ paragraph, 1^st^ sentence).**
o Material and methods, Ocular examination and Imaging, page 5: add the word “also” in the sentence “Fundus photography, OCT, and OCTA were (also) performed on the healthy fellow eyes”.

**Response:** The corresponding sentence has been corrected according to the reviewer’s comment**. (Materials and methods, Ocular Examination and Imaging, 1^st^ paragraph, the last sentence).**

o Material and methods, main outcomes measures and statistical analysis, page 6: replace “for normality tests” with “to test for normality” in “KolmogorovSmirnov and Shapiro-Wilk analyses were used for normality tests.”

**Response:** The corresponding sentence has been corrected according to the reviewer’s comment **(Materials and methods, main outcome measures and statistical analysis, 1^st^ paragraph, 2^nd^ sentence).**
o Material and methods, main outcomes measures and statistical analysis, page 7: replace “the factors” with “variables” in “Binary logistic regression was used to analyze the factors associated with visual outcomes.”

**Response:** The corresponding sentence has been corrected according to the reviewer’s comment **(Materials and methods, main outcome measures and statistical analysis, 1^st^ paragraph, 8^th^ line).**
o Results, page 8: add the word “present” in the sentence “The EIFL was (present) in 7 eyes.”

**Response:** The corresponding sentence has been corrected according to the reviewer’s comment **(Results, 1^st^ paragraph, 3^rd^ sentence).**
o Table 1: it is visually better to present sex and lens status as proportions using percentages instead of solely reporting absolute numbers.

**Response:** A percentage was additionally inserted to Sex and Lens status according to the reviewer’s comment **(4 (17%) / 20 (83%), 21 (88%) /3 (12%); Results, Table 1).**
o Table 1: It should be mentioned what the measures used for the symptom period are i.e. (mean +-SD, range).

**Response:** Type of the measures used was added as the expression of “(median (interquartile range); range)” according to the reviewer’s comment **(Results, Table 1).**

o Table 1: A typo in the word "months" (misspelled as "month") next to “symptom period” should be corrected.

**Response:** The typo in the word “months” was corrected according to the reviewer’s comment **(Results, Table 1).**
o Results section, under “Topographic changes in the Macula”, first sentence, page 9: the terms “the changes in parameters” and “their associations” are ambiguous. It would be helpful to the reader to define what those entail.

**Response:** To be more specific, “parameters” was modified to “topographic parameters including FD, FVs, VFV, DFV, and foveal location” and “their associations” was changed to “their association with CSMT and those of healthy fellow eyes was confirmed.”. **(Results, Topographic Changes in the Macula, 1^st^ paragraph, 1^st^ sentence).**

o Results, page 9, in Fig. 2 legend: “the X coordinate moved to the disc after 6 months” could be replaced with “the X coordinate moved towards the disc after 6 months.”

**Response:** The corresponding sentence has been corrected according to the reviewer’s comment **(Results, Fig 2, 2^nd^ sentence in the legend).**
o Figure 2 legend, page 9: I suggest that the last 2 sentences of C and D be included as part of the results section of the manuscript and not as part of the legend, as they describe findings. I would also just describe what the graphs are about in (A) and (B) and explain the findings in the manuscript.

**Response:** To avoid misleading expressions, the legend in Fig 2b has been modified to “Most of the movement in median foveal location tends to be occurred within 1 month and the X coordinate continuously moved towards the disc for 6 months.”. Details about findings in Fig 2a and 2b are described in results (The foveal center continuously moved towards the optic disc during the follow-up period. The median foveal X coordinates were 153.6 µm, 162.2 µm, and 203.0 µm at 1, 3, and 6 months, respectively, based on the baseline location “0”.) The last 2 sentences of Fig 2c are a summary of the first paragraph of Topographic Changes in the Macula. **(Results, Topographic Changes in the Macula, 1^st^ paragraph)** The last 2 sentences of Fig 2d were included as part of the results section of the manuscript **(Results, Topographic Changes in the Macula, the last paragraph, 2^nd^ sentence)**
o Results, page 10: could replace the word “to” with “towards” in the sentence: “the fovea continuously moved to the optic disc during the follow-up period.”

**Response:** The corresponding sentence has been corrected according to the reviewer’s comment **(Results, topographic changes in the macula, 1st paragraph, 17^th^ line).**
o Results, page 11, figure 3 legend: remove the dash in “blue-cross” and “redcross”.

**Response:** The dashes had been removed according to the reviewer’s comment **(Results, Fig 3, 1^st^ and 3^rd^ sentences in the legend).**
o Results, page 11, figure 3 legend: could remove the last sentence as this was already mentioned in the results and will be reiterated in the discussion.

**Response:** The corresponding sentence has been removed according to the reviewer’s comment **(Results, Fig 3, the last sentence in the legend).**
o Results, bottom of page 11, under Association between topographic factors and visual outcomes: add the word “with” in the sentence: “eyes with a thicker CSMT presented (with) worse BCVA at baseline.”

**Response:** The word “with” has been added to the sentence according to the reviewer’s comment **(Results, Association Between Topographic Factors and Visual Outcomes, 1^st^ paragraph, 1^st^ sentence).**
o Results, bottom of pages 11 and 12, under Association between topographic factors and visual outcomes: “change in BCVA” and “change in metamorphopsia” should be subtitles for better clarity.

**Response:** “change in BCVA” and “change in metamorphopsia” were adopted as level 3 heading (subtitle for level 2 heading “Association between topographic factors and visual outcomes”) according to the reviewer’s comment **(Results, Association Between Topographic Factors and Visual Outcomes).**
o Results, page 13: replace “such” with” “those” in “Such eyes still showed worse vM and hM scores”

**Response:** The word “such” has been replaced with “those” according to the reviewer’s comment **(Results, Association Between Topographic Factors and Visual Outcomes, the last sentence).**

o Discussion, second sentence, page 14: “the fovea moved horizontally to the optic disc” could be replaced with “the fovea moved horizontally towards the optic disc”, implying that it moved nasally, as the results described.

**Response:** The corresponding sentence has been corrected according to the reviewer’s comment **(Discussion, 1^st^ paragraph, 2^nd^ sentence).**
o Discussion, page 14, last sentence of first paragraph can be rewritten as follows: “This study investigated the association between macular topographic changes and visual acuity via multimodal imaging comprising fundus photographs, OCT, and OCTA.”

**Response:** The corresponding sentence has been changed as recommended by the reviewer **(Discussion, 1^st^ paragraph, the last sentence).**
o Discussion, page 15: A typo in the word "INL" (misspelled as "ILN") in the sentence “ILN thickness on OCT” should be corrected.

**Response:** The typo in the word “INL” (misspelled as “ ILN”) has been corrected according to the reviewer’s comment **(Discussion, 4^th^ paragraph, 5^th^ line ).**
o Discussion, page 16: A typo in the word "disc" (misspelled as "disk") in the sentence “more horizontal movement of the fovea to the optic disk” should be corrected.
**Response:** The typo in the word “disc” (misspelled as “disk”) has been corrected according to the reviewer’s comment **(Discussion, 5^th^ paragraph, 2^nd^ sentence).**
o Discussion, page 16: “more horizontal movement to the optic disc” could be replaced with “more horizontal movement towards the optic disc.”

**Response:** The corresponding phrase has been changed as recommended by the reviewer **(Discussion, 5^th^ paragraph, 2^nd^ sentence).**
o Discussion, page 16: “foveal movement to the optic disc” could be replaced with “foveal movement towards the optic disc.”

**Response:** The corresponding phrase has been changed as recommended by the reviewer **(Discussion, 5^th^ paragraph, 18^th^ line).**
o Discussion, page 16: add the terms “in the context of” for better clarity in the sentence: “This reasoning about metamorphopsia have also been suggested (in the context of) macular hole.”

**Response:** The corresponding phrase has been added as recommended by the reviewer **(Discussion, 5^th^ paragraph, the last sentence).**
o Discussion, page 17: can rephrase “Different outcomes can be achieved if eyes with iERM are classified and distinguished based on severity” to “We may also obtain different outcomes if eyes with iERM were classified and distinguished based on severity” for better flow of the paragraph.

**Response:** The corresponding sentence has been changed as recommended by the reviewer **(Discussion, 5^th^ paragraph, the last sentence).**
o In figure 1 (E), the text is not very clear.

**Response:** The blurred letters in Fig 1e are indicators for location used in the customized program, but do not carry any special meaning. It was removed based on the comment. **(Fig 1e)**

o In figure 1, I would make the red and blue crosses more apparent (i.e. more bold and sharper borders). I would also consider changing the color of the red cross to something with higher contrast as red blends in with the color fundus photo’s background.

**Response:** Based on the reviewer's comments, the red and blue cross was processed to be more apparent. **(Fig 1)**
o In figure 3, I would suggest replacing the red dot at the optic disc with another color such a black or green as red might be mistaken as part of retinal blood vessels.

**Response:** Based on the reviewer's comment, it was re-marked as replacing the red dot at the optic disc with green color. **(Fig 3)**

o In figure 3, make the blue cross more apparent in (A) and the red cross more apparent in (B). Again, in (B) and (C ), I would also suggest changing the color of the red cross for the same reason discussed above for figure 1.

**Response:** In Fig 3, based on the reviewer's comments, the red and blue cross was processed with more apparent color and shape (the dot on 3a and 3b, the cross on 3c). In Fig 3, it was re-marked as replacing the red dot at the optic disc with green color. **(Fig 1)**
