# Supplementary material for: Topographic changes in macula and its association with visual outcomes in idiopathic epiretinal membrane surgery
Source: PLoS One. 2025 Jan 9;20(1):e0316847. doi: 10.1371/journal.pone.0316847 (PMC11717306; doi:10.1371/journal.pone.0316847)
Supplement: Attachment — Submitted filename: Response to Reviewers.docx [file pone.0316847.s005.docx]

**Point-by-point response to the reviewer’s comments**

Dear Editor & Reviewers,

We appreciate all suggestions and corrections. All authors have carefully reviewed these comments. Our detailed responses to comments are addressed below.

**Review Comments to the Author**

**Reviewer #1**: Notes:

1. The following sentence included in the Introduction: “A yellow spot on the fovea was also noted in chronic ERM fundus photograph” ; unsure what this means? Or what it means for your project? Would either rephrase/elaborate on it more or take it out all together

**Response:** To characterize the funduscopic features of the epiretinal membrane in the chronic phase, we described "yellow spot on the fovea" as subfoveal pigmentary change. However, this sentence doesn't have any relevance to our study. Therefore, based on the reviewer's recommendation, we deleted it. (Reference: Subfoveal pigment changes in patients with longstanding epiretinal membranes, Gomes NL, et. al., *Am J Ophthalmol*. 2009;147(5):865-868.) **(Fifth sentence of second paragraph in the introduction)**.

2. In the Figure 2 legend would change the following phrase: “Most of the movement in median foveal location tends to be occurred within 1 month” to “Most of the movement in median foveal location tends to occur within 1 month”. It is currently grammatically incorrect

**Response:** We appreciate the reviewer for pointing out the grammatical error and have corrected it from “Most of the movement in median foveal location tends to be occurred within 1 month” to “Most of the movement in median foveal location tends to occur within 1 month”. **(Second sentence of figure 2 legend)**

3. I would address the fact that 88% of pts had concurrent cataract surgery and discuss that this could or could not affect the results in BCVA for these pts compared to the ones that did not have cataract extraction

**Response:** In our discussion, we described limitations cataracts can act as confounding factors as follow: “Although eyes with visually affecting cataract (more than grade 2 nuclear opalescence or cortical cataract) were excluded, mild cataract could be a potential confounding factor in assessing visual acuity.”. In order to provide a supplementary explanation for the limited impact of these limitations on this study, the results showing that opacity below grade 2 in the LOCS III system did not significantly affect visual acuity were added as follows. “However, according to the results of a study on the relationship between nuclear opalescence based on the LOCS III system and visual acuity, nuclear opalescence of grade 2 or lower had little effect on visual acuity decrease. Additionally, cortical cataracts of grade 2 or lower involve only peripheral changes and are unlikely to affect central vision. For these reason, although 88% of the patients in our study showed low-grade cataract changes, it is thought that cataract changes in the eyes included in our study would have had little effect on vision.”

**(Fourth sentence of the last paragraph (limitation), Discussion).**

**Reviewer #2**:

1. Introduction: In the sentence "Previously studied methodologies were hard to figure out confounding variables that might affect prognosis of surgery ", replace " were hard to figure out" with "failed to identify"
**Response:** We appreciate the reviewer for pointing out the grammatical error and ambiguous expressions. The corresponding sentence has been corrected according to the reviewer’s comment **(Third sentence of 3^rd^ paragraph, Introduction).**

2. Results, figure 2 legend: (B) replace "to be occured" in "Most of the movement in median foveal location
tends to be occurred within 1 month" with "occur"
**Response:** We have corrected it from “Most of the movement in median foveal location tends to be occurred within 1 month” to “Most of the movement in median foveal location tends to occur within 1 month”. **(Second sentence of figure 2 legend)**

3. Figure 1 (e): typo in "supeor"; should be "superior"
**Response:** The typo in “supeor” has been corrected to “superior” on (e) panel of figure 1.

4. Results, figure 3 legend: the word "red" should be changed to green since the color of the dot at the disc was changed.

**Response:** After changing the color of the indicator in figure 3, the legend could not be modified in the first revision. According to the reviewer's comment, the red dot on the disc was changed to a green dot.

5. Results, figure 3 legend: (A): the center of the fovea was marked with a blue dot on the OCTA (not a blue cross). Same comment for (B); the fovea was marked by a red dot (not a red cross)

**Response:** After changing the color of the indicator in figure 3, the legend could not be modified in the first revision. According to the reviewer's comment, the blue cross and red cross in (A) and (B) were changed to blue dot and red dot.

6. Discussion: Replace "in particular" with "particularly" in the sentence "The conflicting outcomes of the association between changes in macular topography and visual gain would result from the different methodologies, in particular, the extent of the macula covered by the parameters" and remove the comma after it.
**Response:** The pointed out parts have been corrected as follows. “The conflicting outcomes of the association between changes in macular topography and visual gain would result from the different methodologies, particularly the extent of the macula covered by the parameters.” **(Seventh sentence of 3^rd^ paragraph, Discussion).**

7. Discussion: In the sentence "When the ERM contracts the macula centripetally, the retinal tissues become packed and thickened within the small, contracted macula", replace "the retinal tissues" with "the retinal layers" and replace the word "packed" with "distorted" or "wrinkled"

**Response:** The pointed out parts have been corrected as follows. “When the ERM contracts the macula centripetally, the retinal layers become distorted and thickened within the small, contracted macula” **(Seventh sentence of 5^th^ paragraph, Discussion).**
