# Supplementary material for: Topographic changes in macula and its association with visual outcomes in idiopathic epiretinal membrane surgery
Source: PLoS One. 2025 Jan 9;20(1):e0316847. doi: 10.1371/journal.pone.0316847 (PMC11717306; doi:10.1371/journal.pone.0316847)
Supplement: Attachment — Submitted filename: Reviewer comments plos one .docx [file pone.0316847.s004.docx]

Reviewer comments:

1. The following sentence included in the Introduction: *“A yellow spot on the fovea was also noted in chronic ERM fundus photograph”* ; unsure what this means? Or what it means for your project? Would either rephrase/elaborate on it more or take it out all together
2. In the Figure 2 legend would change the following phrase: “*Most of the movement in median foveal location tends to be occurred within 1 month”* to  *“Most of the movement in median foveal locationtends to occur within 1 month”.* It is currently grammatically incorrect
3. I would address the fact that 88% of pts had concurrent cataract surgery and discuss that this could or could not affect the results in BCVA for these pts compared to the ones that did not have cataract extraction
